# Africa's COVID-19 Situation in Focus and Recent Happenings: A Mini Review

**DOI:** 10.3389/fpubh.2020.573636

**Published:** 2020-12-17

**Authors:** John Elvis Hagan, Bright Opoku Ahinkorah, Abdul-Aziz Seidu, Edward Kwabena Ameyaw, Thomas Schack

**Affiliations:** ^1^Department of Health, Physical Education, and Recreation, University of Cape Coast, Cape Coast, Ghana; ^2^Neurocognition and Action-Biomechanics-Research Group, Faculty of Psychology and Sport Sciences, Bielefeld University, Bielefeld, Germany; ^3^The Australian Center for Public and Population Health Research [ACPPHR], Faculty of Health, University of Technology Sydney, Sydney, NSW, Australia; ^4^College of Public Health, Medical and Veterinary Sciences, James Cook University, Townsville, QLD, Australia; ^5^Department of Population and Health, University of Cape Coast, Cape Coast, Ghana

**Keywords:** COVID-19, intensified surveillance, detection, quarantine, contact tracing, Africa

## Abstract

Given that COVID-19 (SARS-CoV-2) has crept into Africa, a major public health crisis or threat continues to linger on the continent. Many local governments and various stakeholders have stepped up efforts for early detection and management of COVID-19. This mini review highlights the current trend in Africa, history and general epidemiological information on the virus. Current ongoing efforts (e.g., improving testing capacity) and some effective ways (e.g., intensified surveillance, quick detection, contact tracing, isolation measures [e.g., quarantine], and social distancing) of preventing and managing COVID-19 in Africa are described. The review concludes by emphasizing the need for public health infrastructure development (e.g., laboratories, infectious disease centers, regional hospitals) and human capacity building for combating COVID-19 and potential future outbreaks. Additionally, regular public health educational campaigns are urgently required. Future epidemiological studies to ascertain case fatality and mortality trends across the continent for policy directions are necessary.

## Background

The recent novel coronavirus (COVID-19) pandemic has speedily escalated from China to other geographical boundaries, including Africa (1, 2). The initial ill-conceived thoughts that Africa is not conducive for the virus and that Africans have strong immune systems to combat the virus have been debunked with multiple confirmed cases (3). The swift rise of the pandemic across the continent is worrisome and has created a serious public health threat. This mini review provides the history and general epidemiological information on the virus as well as current trends in Africa. On-going concerted efforts and some effective ways of preventing and managing COVID-19 in Africa are also described.

## History and Epidemiological Information on COVID-19

COVID-19 originated from a group of coronaviruses with RNA genome ranging from 60 nanometer [nm]to 140 nm in diameter with spike-like projections sprouting on a crown-like feature under the microscope; hence its coveted name coronavirus. Generally, four specific coronaviruses, namely HKU1, NL63, 229E, and OC43 have been found in human beings to cause mild respiratory infection (4, 5). The pathological characteristics of the current COVID-19 is similar to the severe acute respiratory syndrome (SARS; 2002–2003) and Middle East respiratory syndrome (MERS; 2012) outbreaks. SARS originated through zoonotic transmission of a novel coronavirus from bats through palm civets in markets in Guangdong Province, China from 2002 to 2003. This virus was reported to have affected approximately 8,422 people mainly in China and Hong Kong, with 916 reported deaths (Case Fatality Rate [CFR], 17%) (6). MERS also emerged from zoonotic transmission of a group of viruses that had previously been detected in bats and cultured from respiratory secretions of a patient who had died from SARS in 2012 (7). The same causative organism was previously discovered in clinical trials from a severe occurrence of acute respiratory disease, which was first reported in humans in Jordan in 2012 (8, 9). By 2013, 55 laboratory-confirmed cases of MERS were reported in Jordan, Saudi Arabia, the UK, France, Italy, Germany, and Tunisia (10). According to Wu and McGoogan (7), all the 3 viral contagions commonly manifest with fever and cough, which regularly cause lower respiratory tract disease with poor clinical outcomes related to old age and primary health conditions. The detection of infection requires testing of respiratory tract samples (e.g., throat swabs) through clinical diagnosis which can also be done based on symptoms, exposures, and chest imaging.

Although early genetic assessment on COVID-19 in China revealed that the virus was similar to SARS-CoV and MERS-CoV, scientific evidence in the past months has revealed major differences between the other outbreaks and characteristics of COVID-19 (11). Reflecting on available epidemiological data on the current upsurge, since December 2019 when the first cases were recorded in Wuhan, China, the virus spread has solely been driven by human-to-human transmission and not only as a result of continuous global spillover (11). According to Heymann et al., COVID-19 reproduces in the upper respiratory tract and shows minimal symptoms comparable to the other human coronaviruses that manifest with common colds during winter. Affected persons create a large amount of virus in the upper respiratory tract at the onset or prodrome period, go about normal duties with ease and thus facilitate the spread of the disease. Comparatively, SARS-CoV transmission did not readily occur during the prodromal phase when infected persons were reported mildly ill, and most transmissions were believed to have happened when infected individuals showed severe illness. These symptoms made it easier to control SARS-CoV outbreaks compared to COVID-19 (12). Because the COVID-19 virus is transferred via droplets produced through coughing and sneezing by persons with notable symptoms and in some cases from asymptomatic individuals, virtually all ages are susceptible (13). Infection could be contracted either by inhaling these tiny microscopic droplets or touching surfaces infected by the virus and then touching the nose, mouth, eyes and/or face (13). According to Kampf et al. (14), the virus can stay active on surfaces for days in positive atmospheric conditions but can also be killed within a moment with common antiseptics or disinfectants.

Reported clinical features of COVID-19 range from acute respiratory distress syndrome to organ dysfunctions. Common symptoms also include fever, headache, sore throat, cough, breathing difficulties, and exhaustion. The infection can progress within a week to pneumonia, respiratory failure, and death in different patients (15). According to Singhal (4), the duration from the onset of symptoms to dyspnea is 5 days, about 7 days to hospitalization, and development of acute respiratory distress syndrome (ARDS) in 8 days. Infected persons may require intensive care admission. Reported recovery may take 2 to 3 weeks. Serious consequences and deaths are more pronounced in the elderly and persons with co-morbidities (50–75% of fatal cases) and fatality rate of adult patients ranges from 4 to 11% whereas overall case fatality rate is estimated to range between 2 and 3% (16, 17).

## Recent Covid-19 Trends in Africa

**Figure 1:**
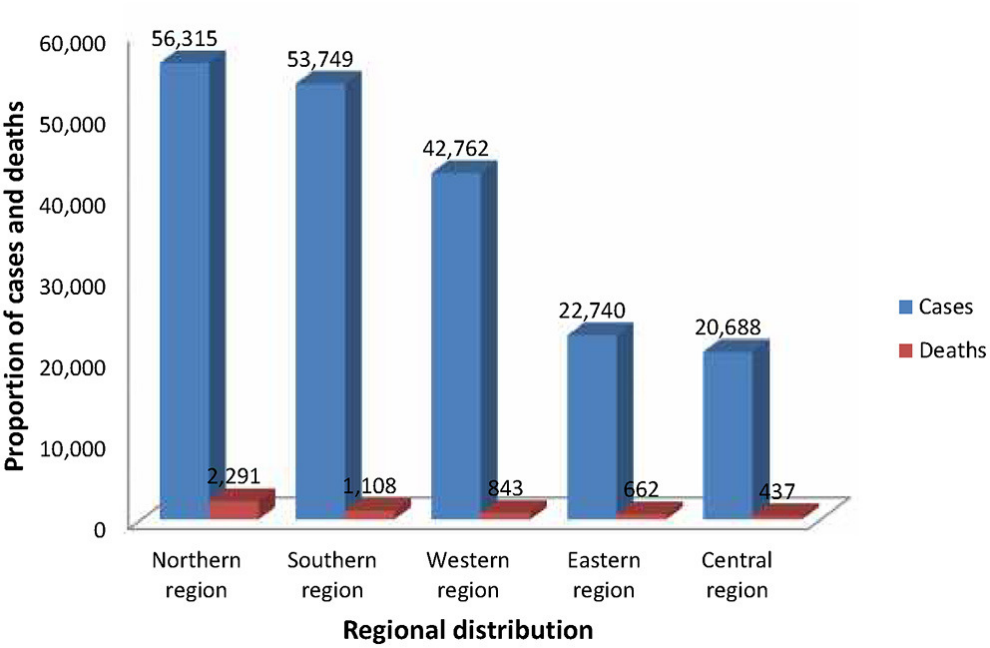
Regional distribution of COVID-19 cases and deaths in Africa as of 9 am EAT 9 June 2020. This figure is original and based on data from Africa Center for Disease Control (4).

Available data shows that since the first case of COVID-19 was noted on the 14th February, 2020 in Egypt, a total of 196,254 COVID-19 confirmed cases and 5,341 deaths (Case Fatality Rate [CFR]: 2.7%) have been reported in 54 African countries as of 9 am EAT 9 June 2020 (18). This figure is an estimation of 2.8% of all cases reported globally. The proportion of confirmed COVID-19 cases and deaths reported by African sub-regions in the order of severity is as follows: Northern region (56,315 cases, 2,291 deaths), Southern region (53,749 cases; 1,108 deaths), Western region (42,762 cases, 843 deaths), Eastern region (22,740 cases, 662 deaths), and Central region (20,688 cases, 437 deaths, see Figure 1).

**Figure 2:**
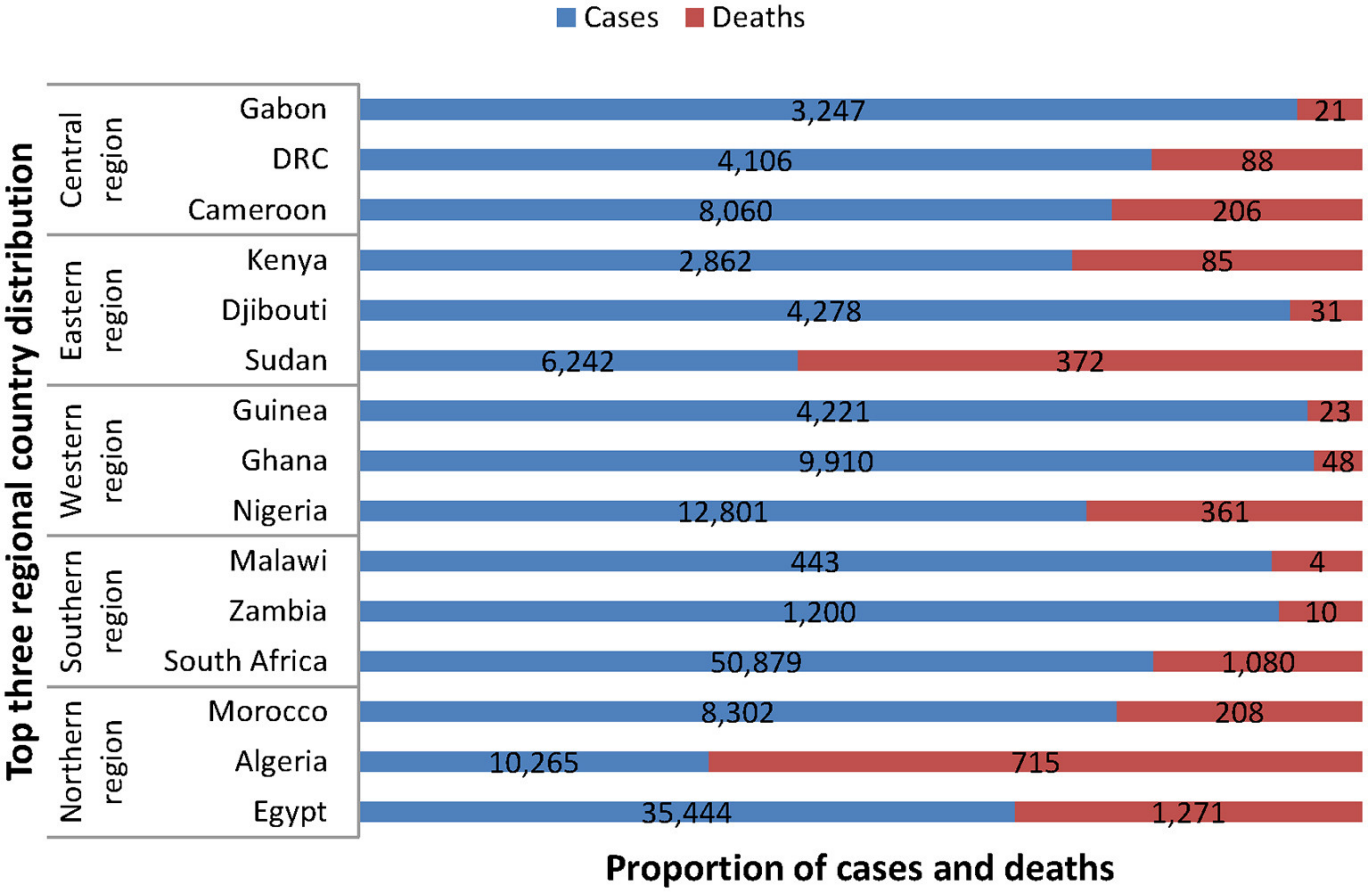
Top three regional country distribution of COVID-19 cases and deaths in Africa as of 9 am EAT 9 June 2020. This figure is original and based on data from Africa Center for Disease Control (4).

The top three countries worst affected in each region as at the beginning of 9th June 2020 9 am. EAT stood as follows: Northern region (Egypt, 35,444 cases, 1,271 deaths; Algeria, 10,265 confirmed cases, 715 deaths; Morocco, 8,302 cases, 208 deaths), Southern region (South Africa, 50,879 cases, 1,080 deaths; Zambia, 1,200 cases, 10 deaths; Malawi, 443 cases, 4 deaths), Western region (Nigeria, 12,801 cases, 361 deaths; Ghana, 9,910 cases, 48 deaths; Guinea, 4,221 cases, 23 deaths), Eastern region (Sudan, 6,242 cases, 372 deaths; Djibouti, 4,278 cases, 31 deaths; Kenya, 2,862, 85 deaths), and Central region (Cameroon, 8,060 cases, 206 deaths; Democratic Republic of Congo [DRC], 4,106 cases, 88 deaths; Gabon, 3,247 cases, 21 deaths, see Figure 2) (18).

**Figure 3:**
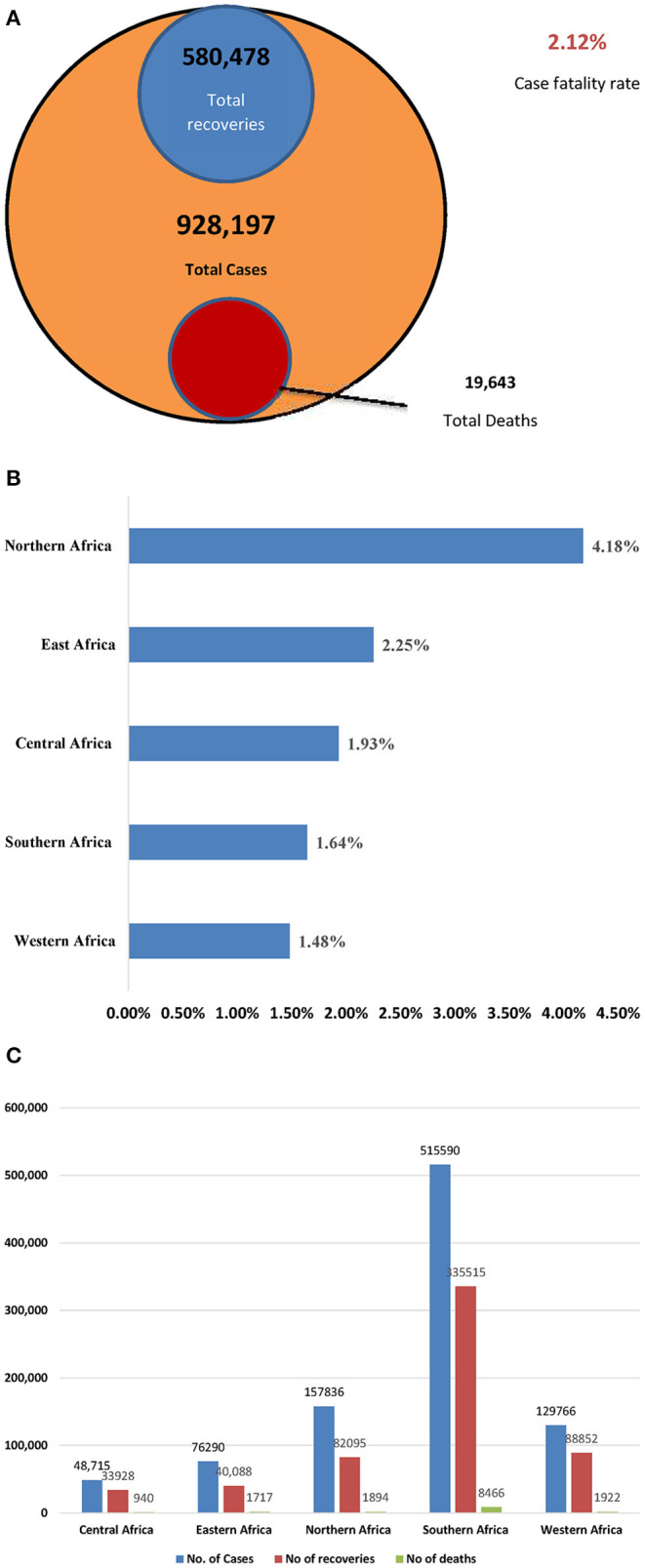
**(A–C)** Epidemiological metrics in Africa by 31st July, 2020 (20). **(A)** Total cases, recoveries, deaths, and total fatality rate. **(B)** Case fatality rate by region. **(C)**. Epidemiologic situation by region.

By 4th August, 2020, a total of 968,020 COVID-19 cases and 20,612 deaths (CFR: 2%) had been reported in 55 African countries. The estimation of new COVID-19 cases reported by region still represented 5% of all cases globally. Below are the proportions of cases by sub-region: Southern region 65% (70,318), Northern region 12% (13,016), Western region 9% (9,638), Eastern region 11% (12,367), and Central region 3% (3,444). A total of 6 countries accounted for 80% of the new COVID-19 cases reported within the period in a descending order: South Africa (59%), Morocco (5%), Algeria (4%), Kenya (4%), Ghana (4%), and Ethiopia (4%). South Africa (890), Djibouti (524), Sao Tome and Principe (437), Cape Verde (431), and Gabon (364) reported the most cumulative COVID-19 cases per 100,000 in Africa. Reported case fatality rates comparable to or higher than the global case fatality rate of 4% were noted in 11 African countries; Chad (8%), Sudan (6%), Liberia (6%), Niger (6%), Egypt (5%), Mali (5%), Burkina Faso (5%), Angola (5%), Algeria (4%), Sierra Leone (4%), and Tanzania (4%) (19). The pictorial epidemiological information of COVID-19 per region as of 31st July, 2020 is captured in Figures 3A–C below (20).

Africa CDC brief report on public health and social measure implementation indicated that these new cases decreased by 23% between 28th July and 10th August while new deaths increased by 11% within the same time period. Total COVID-19 cases exceeded 1 million for the first time which was cited as the peak period. The decrease in new cases was attributed to South Africa, which recorded a 35% decline in newly reported cases, with data showing that the COVID-19 outbreak might have peaked in some provinces or districts that were earlier affected by the virus. Though South Africa still leads in new cases, Morocco, Kenya and Ethiopia reported rising case counts and new deaths. The number of tests performed per positive case has remained low (6 tests per case, see Table 1), suggesting that many cases are undetected. With the low tests per case ratios in many African countries (e.g., Algeria, DRC, Egypt, Madagascar, Somalia, South Sudan and Sudan), denoting poor testing capacity, indications are that many cases on the continent are less likely to be detected. Therefore, reported decreases in new cases ought to be interpreted with caution in many countries. The month of October saw a slight increase in the number of COVID-19 cases per 100,000 population. Again, 6 countries accounted for nearly 84% of the new COVID-19 cases; Morocco (31%), South Africa (18%), Libya (11%), Tunisia (9%), Kenya (7%), and Ethiopia (6%) by 27th October, 2020 (19).

**Table 1:** Africa COVID-19 Situation between late July and Early August 2020.

**Africa Union Region**	**Total Death/Trend 28 July−10 August^a^**		**Total Deaths/Trend 28 July−10 August^a^**		**Countries where Test per cases^b^ <10**
Central	50353	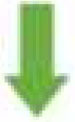	961	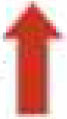	Cameroon **(9)** Central African Republic **(6)** Chad **(9)** Congo **(6)** DRC **(5)** Equatorial Guinea **(9)** Sao Tome & Principe **(7)**
Eastern	91045	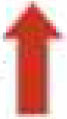	2007	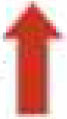	Comoros **(6)** Madagascar **(4)** Somalia **(6)** S. Sudan **(6)** Sudan **(3)**
Northern	177118	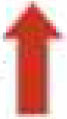	7126	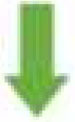	Algeria **(4)** Egypt (5)
Southern	589343	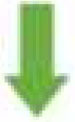	11093	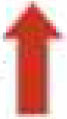	Eswatini **(9)** Malawi **(7)** South Africa**(6)**
Western	140601	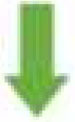	2087	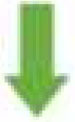	Cote d'Ivoire **(6)** Gambia **(7)** Guinea **(8)** Guinea Bissau **(6)** Nigeria **(7)**

a *The total number of cases reported is the number of cases reported since the start of the epidemic. The trend compares new cases and new deaths during 28 July−10 August to 14–27 July to the previous 2-week period (14–27 July). The trend is illustrated in by an arrow icon: a green arrow indicates a decrease >5%, gray is a decrease/increase within 5%, and red means an increase >5%*.

b *The test per case is the number of tests performed per positive case. Countries with a low number of tests per case (<10) may not be testing widely enough to find all cases. Africa CDC recommends 10–30 tests per case, as a benchmark of adequate testing (19)*.

Given that accurate mortality metrics are essential for assessing the risks and severity often associated with epidemic outbreaks like COVID-19, it is crucial to better understand these associated concepts unambiguously in the estimation of the current pandemic. The generally used metrics during infectious disease outbreaks are the case fatality rate (CFR), case fatality ratio, and case fatality risk, often used interchangeably. Fatality *rate* means a change in deaths per unit time; *risk* denotes an individual probability; whereas *ratio* represents a fraction of two numbers, usually of populations. Therefore, CFR commonly represents the ratio of the total estimated number of deaths to date, *D*(*t*), to the estimated number of all confirmed cases to date *N*c (*t*) or an estimation of the number of deaths to date divided by the estimated total number of confirmed infected cases to date. Hence, these figures could be crucial for the estimation of the COVID-19 disease severity in Africa (21–23).

Conventionally, the reverse transcription polymerase chain reaction (RT-PCR) and antibody tests are used to confirm SARS-CoV-2-positive patients based on population data. To establish accuracy *D*(*t*), the number of patients who actually die of COVID-19 should be appropriately be quantified. For instance, in Italy, deaths of patients with positive RT-PCR testing for SARSCoV-2 are reported as COVID-19 deaths and there could be similar cases across many countries. Therefore, the central criteria for COVID-19-related deaths as a function of CFR are currently not clearly defined and may vary from region to region (24). Other mortality metrics like case fatality risk, denoted as the probability of death of an individual confirmed case within a period of time and infection fatality ratios (IFR), representing the number of deaths to date divided by the number of all infected individuals add to the confusion. For example, IFR = *D*(*t*)/*N*(*t*) requires an estimate of *N*(*t*), the number of total (including unconfirmed) infected individuals [see (21) for detailed description].

According to Böttcher et al. (21), the further spread of SARS-CoV-2 in different countries, including those in Africa, makes it imperative that data on individual cases of death and recovery are easily stratified according to some specific COVID-19 mortality indices such as demographic and population heterogeneity, incubation period, health condition and a patient time of infection before confirmation is made on mortality estimates. Additionally, under-testing also confounds accurate estimation of actual causes of death. For instance, infected individuals who are untested in a given population consist of an unknown population which adds to deaths and recovery, and should be considered as “true” mortalities.

## Current Covid-19 Efforts in Africa and the Way Forward

The erratic COVID-19 increase and associated deaths in Africa underscore the essence for much attention, especially considering the weak public health systems of most African countries (19). Given the inherent challenges with general infrastructure network, poor transportation, neighborhood characteristics (e.g., over-crowding related issues) and inadequate trained health workers in many African countries, pragmatic measures have targeted preventive measures and effective management of confirmed cases. In a quick response to the current upsurge of the pandemic across Africa, the Africa Centers for Disease Control and Prevention in collaboration with WHO African Region instituted an Africa Task Force for Novel Coronavirus that recently launched the “Partnership to Accelerate COVID-19 Testing (PACT): Test, Trace, Treat in Africa” on 4th June, 2020 (18) to increase testing capacity in member states. PACT is to offer the needed support as joint continental strategy to assist member states limit COVID-19 transmission, through strategies such as staff training. As a result, a team of experts, community workers, supplies and other resources have been organized to help test, trace and treat COVID-19 cases in a timely manner to lessen the effect of the pandemic on the African continent. Key strategies such as an expansion of testing to sub-national, research, academic and private laboratories, increasing human resource capacity, reinforcing specimen collection, computerization of testing technologies, and use of pooled sample testing were endorsed (25).

Till date, over million test kits and several thousands of community health workers have been trained (i.e., with knowledge and key skills) and deployed. Again, 80 surveillance rapid responders for COVID-19 have been used in Africa. Additionally, 625,000 Polymerase Chain Reaction (PCR) tests have been advanced to 51 member countries and an extra support of 6,600 GeneXpert cartridges have also been advanced to three member states (i.e., Comoros, Guinea, Sao Tome) that have limited or no capacity for PCR testing. There has been extra supply of pathogen genomics equipment and reagents to member countries except Egypt (18). Laboratory testing capacity has increased from two to forty-four countries on the continent (26). Some countries (e.g., South Africa, Kenya, Morocco, Ghana, Uganda, Ethiopia) have made steady progress with testing capacity from a few hundreds to thousands. By August 2020, nearly 10 million tests have been done on the continent with 11% positivity rate. However, significant sub-regional variations exist in the testing capacity, with the Central Africa region contributing only 3.4% of the total conducted tests, showing that many countries on the continent are still struggling to increase testing capacity. Due to the limited testing capacity across many countries in the African region, pooled sample testing have been recommended to maximize the use of scarce test kits and other supplies to facilitate expansion in testing (27). Considerable efforts are currently ongoing to increase diagnostic capacity across the continent. For example, Morocco currently has 44 hospitals with 32 specialized centers that are fully equipped in response to the pandemic (28). South Africa, Ghana, Nigeria, Algeria, Senegal, and many African countries have laboratories for within country testing of COVID-19. Another testing strategy introduced by some countries (e.g., Ghana) is the Rapid Antibody Tests (RAT). RAT uses a lateral flow technology, similar to home pregnancy tests using nasal swabs for point-of-care diagnosis of COVID-19 by measuring viral antigens or anti-viral antibodies, and results obtained within 10 min after sampling (29, 30). This lateral flow kits against COVID-19 have been developed and are either serology based (detecting host antibodies), or in some cases, antigen based (detecting specific viral proteins) (27). Many countries have created isolation and quarantine centers for the disease, with considerable efforts toward effective contact tracing of potential contacts with infected persons (31).

Besides all the instituted regional interventions, individual member states have adopted other mitigating strategies to reduce the spread of the virus at the national and community level. Since no specific drugs or vaccines are currently available, and health systems are overstretched, many affected countries adopted coercive and non-coercive community interventions with public engagement (32–35). Within local communities, continuation of some mitigation strategies to compel people to avoid crowded places or minimizing crowd sizes and exposure to body contacts (i.e., physical/social distancing) have been implemented. Such interventions included controlling essential social gatherings (e.g., funerals, church services, school attendance, remote working). Essential activities such as schooling and working were done with alternate arrangements such as remote or distance learning. New workplace interventions with work shifts and/ or rotational scheduling to reduce social density against the propagation of the virus have been employed. Conference calling and video conferencing helped working staff and tertiary students adhere to social distancing measures. Other activities such as attending night clubs, music festivals, cultural celebrations, and parties were temporarily suspended but have now been partially lifted with some restrictions (e.g., limited capacity, wearing of face/nose masks, physical distancing). Entertainment through virtual concerts with limited performers or artists has been introduced. Less-essential travels to places with ongoing transmission have been controlled. Since travel bans might trigger fear and affect economic life of the people, home delivery services of essential commodities have been adopted and still being encouraged, especially in the cities. Symptomatic individuals have been supported by telephone or online health consultation. Severe cases have been managed with the provision of essential life support such as oxygen supplies, and mechanical ventilators (32–36).

Adopting and compliance to low-cost evidence-based preventive measures in many African countries where socioeconomic status of the masses is low have been useful. For example, the use of locally made face or nose masks with non-medical cloth made from local fabric has been worn by people at public places and social gatherings. Senior public officials have been seen wearing these masks in public places to serve as an example for the local populace. Alcohol-based hand rub solutions have also been deployed at strategic locations (e.g., transport stations, market places, school/work environment, supermarkets) without restrictions (36). Continuous creation of awareness on the preventive and management measures related to COVD-19 through the media (e.g., radio, television in local languages and dialects) by public health officials and other properly trained analogous personnel across individual countries are still on-going on the African continent.

Protecting local jobs and employees, assets, technology, and infrastructure of critical sectors of economies have also been given a priority. African governments have tried protecting the livelihoods of citizens by preventing unemployment risks during this period. Private companies have been given financial assistance, tax and electricity waivers to maintain continuous production while observing preventive measures. Strategies to minimize workplace transmission have included daily pronouncements of being symptom free by all staff members, and where applicable, requested the screening of staff members. Therefore, screening everyone at a work place before resumption of work could be encouraged, especially in higher risk sectors like the hospitality industry (tourists and hotels), education, aviation or others with high risk of person-person interactions. Other areas such as occupational health service delivery should be crucial for the manufacturing and construction industries by offering close surveillance on workers and possibly test as well as facilitate the quarantine of any member with symptoms pending test results (37).

With the likelihood of reduction in importation during the current crisis, various local markets on the continent might significantly depend on domestic production, including agricultural products. Hence, support for local farmers through the provision of inputs such as fertilizers, weedicides, pesticides, outboard motors, fishing nets as well as mass spraying exercises on local farms could be part of policy interventions for food security. Local governments should provide social welfare interventions through the provision of food and other essential items for personal use, soap and shampoo, to persons living on the fringes of life during the crisis because of restrictions. These support mechanisms will help sustain families' income, preserve the productive capacity of the working population and human capital of enterprises as well as the overall economy of African countries (38). Amidst COVID-19, the entertainment industry should still be encouraged to use innovative virtual performances for the masses to help promote their psycho-social needs. Although country-specific data on health infrastructural expansion are not readily available, there are reported cases of on-going developmental projects (e.g., new disease centers, hospitals, ICUs) in the health sector across many countries (e.g., Morocco, Senegal, Kenya, Ghana, South Africa, Nigeria) through foreign aid to increase health facility capacity and recruitment/ training of healthcare professionals (39).

There are elements of fear, worry and panic about local transmissions and multiple infections because some individuals (e.g., internet bloggers, social commentators, opinion leaders, political officials) are disseminating diverse misinformation or unsubstantiated malicious information on the virus (37–39). This ill-advised COVID-19 related misinformation can rapidly spread the disease and can cause xenophobia on the continent (40–42). The distress, apprehension and other untruths about COVID-19 may have serious effects on disease control, and prior SARS and Ebola outbreaks are clear instances (43, 44). To manage this challenge, individual governments and media institutions should engage public health experts to dominate the media landscape, especially those from the Centers for Disease Control to accurately provide relevant COVID-19 information to avoid fear among the general public (45). Mian and associate reiterated that if health institutions effectively manage public worries through regular education, the level of skepticism among populations often stirred by some social commentators, political opponents, and internet bloggers would be minimized. Local communities, civil society, media as well as other support groups are encouraged to provide accurate COVID-19 information. This goal can be achieved through strategic partnerships at community level so that authorized information is distributed (46).

Negative attitudes of some persons toward formulated public guidelines could be minimized by regular community monitoring by local task force teams. There is also the need to prioritize some special groups (e.g., the disabled, incarcerated persons, people living on the streets, illiterates and other marginalized groups [e.g., mentally challenged persons]) on the continent that are often left to live on the fringes during developmental issues. Considering the ailing health systems of most African countries, existing structures are unable to serve the needs of these groups adequately. Admittedly, these groups could also be at serious risk in times of pandemic outbreaks such as the COVID-19 due to their vulnerability. The COVID-19 precautionary messages, both text and audio, ought to be translated into all predominantly spoken local languages across the continent. The earlier audio-visual aids and braille versions of all precautionary measures are adopted, the better it will be for the continent to ensure the safety of these groups.

## Conclusions

Many countries in Africa have instituted interventions to curtail COVID-19. However, these measures are not without challenges. Many health care systems in Africa are woefully inadequate characterized by under-resourced facilities. The World Health Organization (WHO) and donor agencies should partner local governments in the African region to establish new health infrastructure (e.g., referral laboratories) and equip existing ones with appropriate materials and requisite health logistics. African governments should adopt appropriate context-specific strategies that fit their contextual and geopolitical situations. To effectively deal with public health challenges like the current outbreak, training more human capacities in areas such as surveillance, rapid epidemic response, diagnostic testing, and crisis management should be compelling for governments as well as their private sector and international partners (e.g., WHO, UNICEF, IMF, World Bank). Besides, collaborating with local stakeholder groups (e.g., telecommunication companies- radio/television stations, religious and educational institutions) for regular public health educational campaigns to support the dissemination of COVID-19 information regarding prevention and control practices and creating awareness at the grassroots level are still required.

Although case-fatality rate (CFR) for COVID-19 on the African continent is lower than the global estimation, the scientific evidence about the virus could be inconclusive and may still evolve. Therefore, it is more likely other potential parameters (e.g., population heterogeneity- density, age distribution) may be unraveled in the months to come relative to other non-communicable diseases. It is imperative that compromised healthcare systems, including inadequate human capacity in Africa effectively should be managed effectively to overcome the current outbreak as well as future unforeseen ones. Future epidemiological studies to ascertain case fatality and mortality trends are warranted for policy directions.

## Author Contributions

JH, BA, A-AS, and EA conceived the work. JH, BA, A-AS, EA, and TS wrote and drafted the manuscript. All authors read and approved the final version of the manuscript.

## Conflict of Interest

The authors declare that the research was conducted in the absence of any commercial or financial relationships that could be construed as a potential conflict of interest.

## Abbreviations

CFR: Case Fatality Rate
WHO: World Health Organization
IMF: International Monetary Fund
UNICEF: The United Nations Children's Fund.
